# Pictorial review of the post-operative cranium

**DOI:** 10.4102/sajr.v27i1.2684

**Published:** 2023-07-21

**Authors:** Varsha Rangankar, Anmol Singh, Sanjay Khaladkar

**Affiliations:** 1 Department of Radiodiagnosis, Dr. D. Y. Patil Medical College, Hospital and Research Centre, Dr. D. Y. Patil Vidyapeeth, Pune, India

**Keywords:** cranium, craniotomy, craniectomy, complications, post-operative, Trephine syndrome, tension pneumocephalus, paradoxical herniation

## Abstract

Imaging evaluation of the brain and cranium after cranial surgery is a routine and significant part of the workflow of a radiology department. Various normal expected findings and early and late complications are associated with the post-operative cranium. In this pictorial review, the authors describe the typical imaging features of the spectrum of various conditions associated with cranial surgery with illustrative cases.

**Contribution:**

A good knowledge and understanding of the spectrum of imaging appearances in the post-operative cranium is vital for the radiologist to accurately diagnose potential complications and distinguish them from normal post-operative findings, improving patient outcomes and guiding further treatment.

## Introduction

Surgical procedures like burr holes, craniotomy and cranioplasty are commonly performed for various neurosurgical indications such as intra-axial and extra-axial haemorrhages and collections, tumours, and infections.^1^ Imaging, especially CT, is often performed in the post-operative follow-up of these patients. When interpreting these scans, it is imperative to be aware of the operative procedure performed and the expected post-operative findings, such as pneumocephalus, routine post-operative inflammatory changes and haemorrhages. At the same time, radiologists need to be vigilant of the abnormal imaging findings and be able to identify the various associated post-operative complications such as infections, tension pneumocephalus, sinking flap and herniation.

CT is the first-line modality for these cases, because of short scan times and accessibility, and is routinely used for post-surgical follow-up. While MRI is better than CT for certain conditions such as infection, intracranial collections and ischemia, it is often limited by incompatibility or artifact related to surgical material and implants. This article discusses the surgical techniques with expected imaging findings and describes the important post-operative complications.

## Neurosurgical techniques and imaging appearances

### Burr holes

**FIGURE 1:**
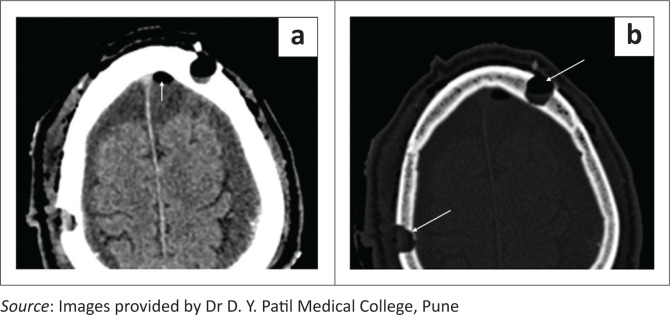
Axial soft tissue (a) and bone window (b) CT head images showing burr holes, seen as rounded well defined defects in the skull bones in the left frontal and right parietal regions (long white arrows). Note the small postoperative pneumocephalus in the left frontal region (a, short white arrow).

Burr holes are small holes that are made in the skull bones using a surgical drill. The usual indications include insertion of a device such as a ventricular drain, endoscope or a deep brain stimulator electrode, drainage of a subdural haematoma and provision of access for stereotactic brain biopsy.^1^ On CT, a burr hole appears as a focal well-defined defect in the calvarium (Figure 1). In the acute setting, associated subgaleal or extradural fluid collections and tiny air foci may be seen.^2^

### Craniotomy

**FIGURE 2:**
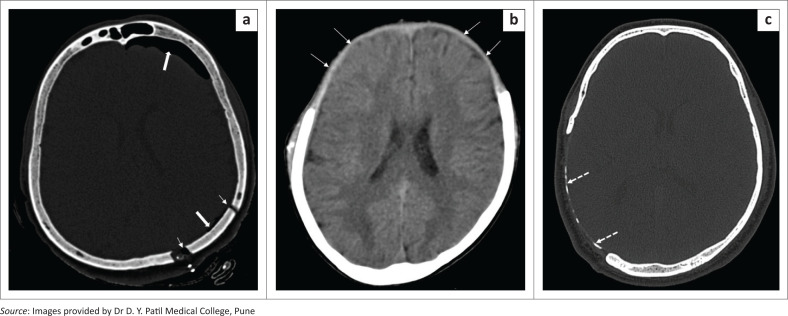
Axial bone window CT brain image (a) demonstrates a left parietal craniotomy (short white arrows) in a patient who underwent surgery for a left parietal space occupying lesion. Note the post-operative pneumocephalus in the left frontal and parietal regions (thick white arrows). Axial soft tissue window CT brain image (b) reveals bifrontal craniectomy with a normal meningogaleal complex seen as a slightly hyperdense linear structure separating the subcutaneous layer and the subarachnoid space (white arrows). Axial bone window CT image in another patient (c) indicating a right parietal craniectomy with chronic calcifications of the meningogaleal complex (dashed arrows).

Craniotomy is the surgical removal of a segment of the calvarium to achieve neurosurgical exposure (Figure 2a). The calvarial segment is replaced at the end of the procedure. The various types of craniotomies include fronto-spheno-temporal, sub-temporal, anterior or posterior parasagittal, median suboccipital and lateral suboccipital.^3^ The margins of the bone flap are sharp and well defined, with a tram track appearance on CT images in the early post-operative period. Later on, the edges become smooth and rounded as the flap undergoes resorption and remodelling.^4^

### Craniectomy

In contrast with craniotomy, the surgically removed segment of skull bone is not replaced at the end of the procedure in craniectomy (Figure 2). Usual indications are decompression of intracranial contents and removal of an infected bone flap.^5^ The bone flap is usually stored in an abdominal subcutaneous pocket for subsequent cranioplasty. Post-craniectomy, the subgaleal space is usually obliterated, with formation of a meningogaleal complex, which consists of the galea aponeurotica, connective and fibrous tissue, and the duramater (Figure 2b). On CT, this meningogaleal complex manifests as a thin, smooth, slightly hyperattenuating layer that enhances mildly on post-contrast images.^6^ In the chronic phase, calcifications of the meningogaleal complex are commonly seen (Figure 2c).

### Cranioplasty

**FIGURE 3:**
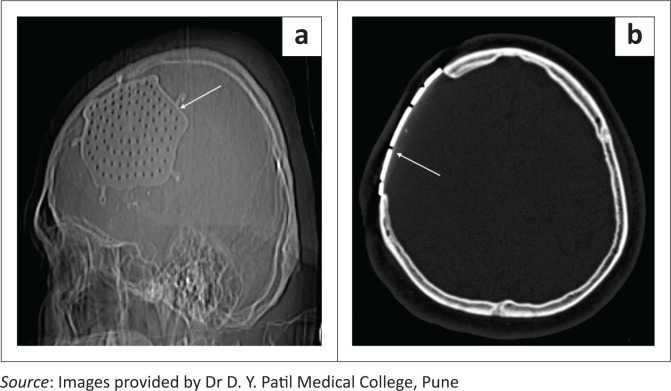
(a, b) CT scanogram and axial bone window CT image demonstrating a right fronto-parietal craniectomy with a titanium cranioplasty implant (long white arrows).

Cranioplasty is a surgical procedure performed to reconstruct a patient’s skull, usually following a previous surgical intervention; or less commonly, in the setting of a congenital defect or after trauma.^7^ The goal of the procedure is to protect the brain from mechanical damage and improve cosmesis. Ideally, the cranioplasty implant should be biocompatible, strong and durable to resist deformation, radiolucent to allow clean visualisation of the underlying brain tissue on CT and also MRI compatible.^7^ Commonly used materials for cranioplasty include autologous bone grafts and synthetic materials such as polymethyl methacrylate, titanium (Figure 3), or ceramics.

## Post-operative findings and complications

### Post-operative haemorrhages

**FIGURE 4:**
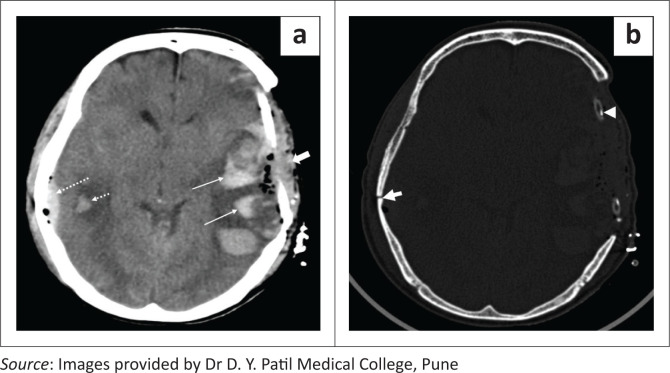
Axial soft tissue window CT brain image (a) in a patient with left decompressive fronto-parietal craniotomy reveals subgaleal hemorrhage (thick short white arrow) with postoperative pneumocephalus and multiple hyperdense intraparenchymal hemorrhages in the left fronto-parietal region with surrounding oedema (long white arrows). Post-operative drain tube is noted at the craniectomy site on image b, bone window CT image (arrowhead). Also note the small lentiform epidural haemorrhage and intraparenchymal hematoma on the right side (a, white dashed arrows) associated with a fracture of the right parietal bone (b, short thick arrow).

**FIGURE 5:**
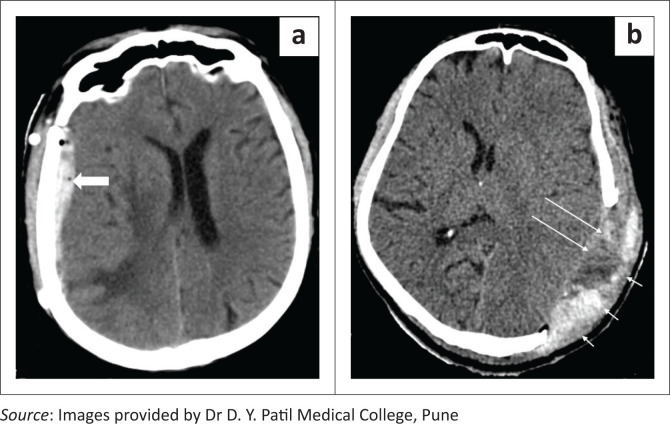
Axial soft tissue window CT image (a) in a patient with a right fronto-parietal craniotomy demonstrates a lentiform hyperdense extra-axial hematoma (thick white arrow) in the right fronto-parietal region with mass effect causing effacement of the right lateral ventricle and midline shift toward left side. Axial soft tissue window CT image (b) in another patient with a left parietal craniectomy reveals a lentiform mixed attenuation epidural hematoma (long arrows) and subgaleal hematoma (short arrows) at the craniectomy site, causing mass effect and resultant effacement of the left cerebral sulci and lateral ventricle with midline shift to right side.

Small scalp and extradural haemorrhages are relatively common and benign findings in the post-operative period. Only about 1% of post-craniotomy intracranial haemorrhages require surgical intervention.^8^ The most common post-operative haematomas are intraparenchymal (43%) (Figure 4), extradural haematoma (33%), subdural haematoma (5%) and mixed (8%) (Figure 5).^8^

Extradural haematomas are situated between the dura and the inner table of the skull. The majority of the post-operative extradural hematomas are regional, in the location related to the surgical site.^9^ Further subtypes are adjacent haematomas that occur at the margins of the craniotomy site and remote extradural haematomas, located distant from the craniotomy site.^10^ The majority of intraparenchymal haemorrhages are small (less than 3 cm in size) and do not cause much neurological compromise. Large haematomas are associated with poorer outcomes.^1^ Causes of large intraparenchymal haemorrhages include poor haemostasis, excessive brain retraction, hypertension in the post-operative period and bleeding disorders.

### Infections

**FIGURE 6:**
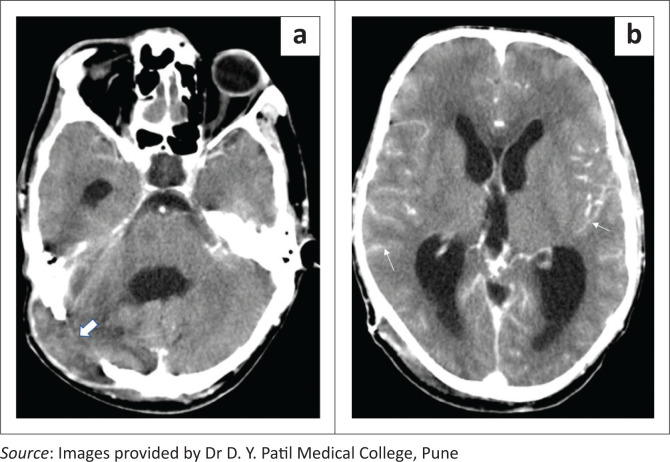
Meningitis. Axial post-contrast soft tissue window CT image in a patient with a right occipital craniectomy (a, thick white arrow) indicates extensive leptomeningeal enhancement (b, thin white arrows). Herniation of the cerebellar parenchyma is also seen at the occipital craniectomy site.

Post-craniotomy infections are a relatively uncommon complication, with an incidence of less than 1% described in the literature, and usually present as extradural abscesses, meningitis (Figure 6), subdural empyema and parenchymal abscesses.^11,12^ The infection usually begins in the subcutaneous plane at the surgical site and extends to the deeper tissues. Contrast imaging plays an important role in diagnosing involvement of the bone flap, extra-axial cerebrospinal fluid (CSF) spaces, meninges and brain parenchyma.

Bone flap infections account for nearly half of all post-craniotomy infections^12^ and usually present 1–2 weeks after surgery. Risk factors include intra-operative breach of the paranasal sinuses, presence of an active infective focus during the surgery, surgery performed for traumatic injuries, longer surgical duration, re-operation, immunodeficient status and post-procedure CSF leakage.^13^ Detached bone flaps are also more likely to get infected as they lack a vascular supply.

**FIGURE 7:**
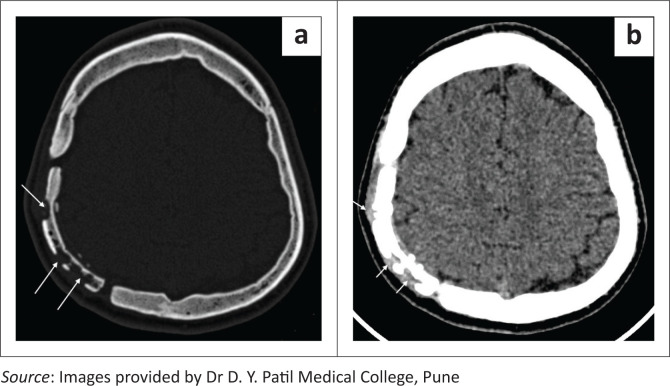
Bone flap infection. Axial bone window CT image in a patient with a right parietal craniotomy demonstrates a mildly sclerosed bone flap with multiple well defined lytic areas (a, long white arrows) and a small adjacent soft tissue component (b, short white arrows).

**FIGURE 8:**
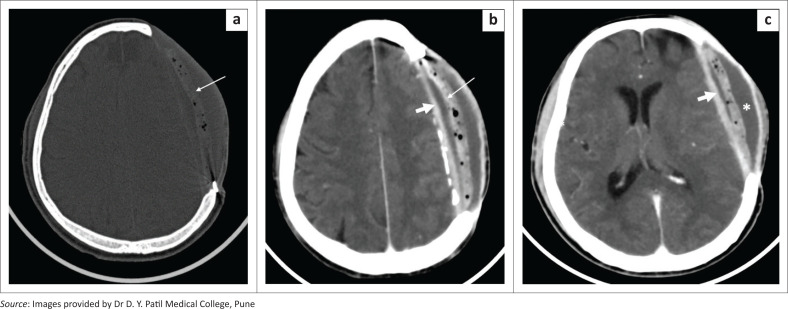
Cranioplasty flap infection. Axial bone window (a) and contrast-enhanced soft tissue window CT images (b, c) in a patient with a left frontoparietal craniectomy reveals an acrylic cranioplasty implant with small air foci (a, b, long arrows). Adjacent epidural (b, c, thick arrow) and subgaleal (c, asterisk) fluid collections along with thickening and enhancement of the meningogaleal complex is seen.

On CT, the bone flap may show an irregular outline with multiple lytic areas or sclerosis of the bone flap (Figure 7). Presence of superficial skin thickening, fat stranding and subgaleal and extradural fluid collections in the presence of bony changes increases the specificity for the diagnosis (Figure 8). On MRI, the marrow of the involved bone appears hypointense on T1 weighted images and hyperintense on T2 weighted images, with diffusion restriction.

**FIGURE 9:**
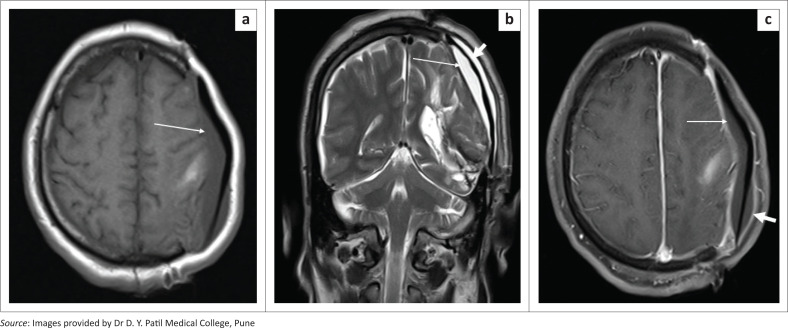
Axial T1 (a), coronal T2 (b) and axial contrast enhanced T1 fat saturated (c) images reveal a left fronto-parietal craniectomy with a T1 hypointense and T2 hyperintense extradural infected collection (long arrows) with thickened, enhancing dura on the post contrast image. A small subgaleal collection (b, c, short arrows) with peripheral enhancement can also be seen.

Extradural abscesses present as biconvex fluid collections, usually adjacent to the craniotomy site, with thickened enhancing dura on contrast enhanced images (Figure 8 and Figure 9).^14^ Subdural empyemas present as extra-axial crescentic fluid collections along the cerebral convexity.^14^

**FIGURE 10:**
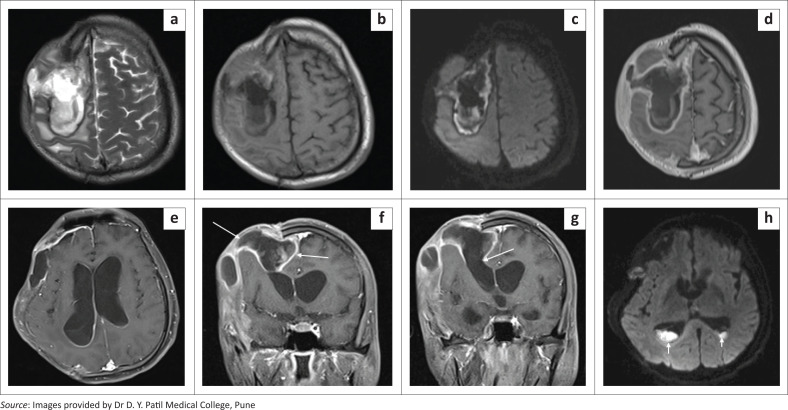
Axial T2 (a) and T1 (b) images show a subdural collection with diffusion restriction (c) in the right fronto-parietal region with parenchymal extension. Post contrast axial (d, e) and coronal (f, g) T1 images show thick, peripheral rim enhancement and extension into the right lateral ventricle (thin white arrows). Axial diffusion weighted image (h) reveals restricted diffusion in the dependent debris within the occipital horns of the lateral ventricles (short white arrows). Apparent diffusion coefficient (ADC) maps not included.

Large subdural empyemas may be associated with subfalcine herniation, significant effacement of the ventricles and sulci and cerebral oedema. These collections are hypointense on T1 weighted images, hyperintense on T2 weighted images and mildly hyperintense to CSF on fluid attenuated inversion recovery (FLAIR) images. They may show diffusion restriction and peripheral rim enhancement (Figures 10a–f). Rarely, the collection can extend into the brain parenchyma and intraparenchymal abscesses can rupture into the ventricle leading to ventriculitis (Figures 10g and h).^15^

### Extracranial herniation

**FIGURE 11:**
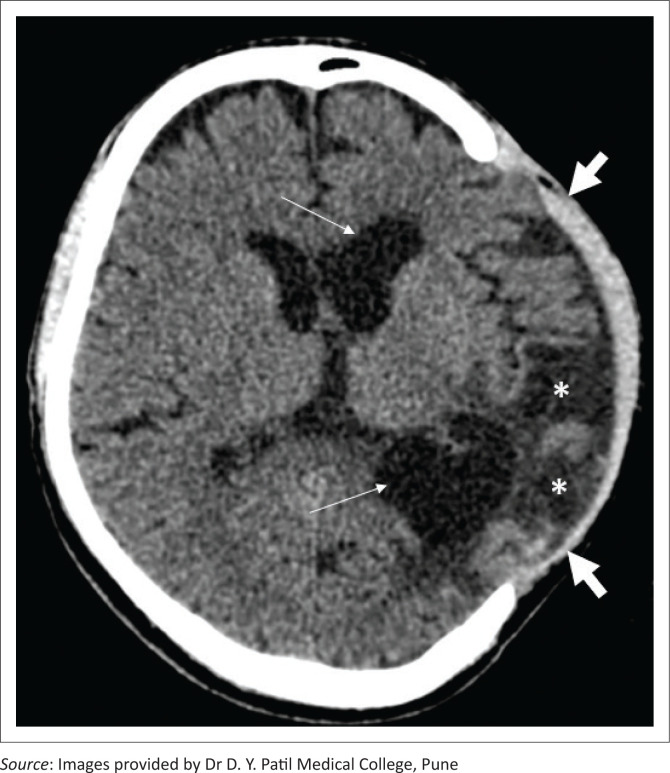
Axial soft tissue window CT image in a patient with a left fronto-parietal decompressive craniectomy indicates herniation of the brain parenchyma through the craniectomy defect (thick white arrows). Extensive encephalomalacia (asterisks) in the left fronto-parietal region along with ex vacuo dilatation of the left lateral ventricle is also seen (white arrows).

**FIGURE 12:**
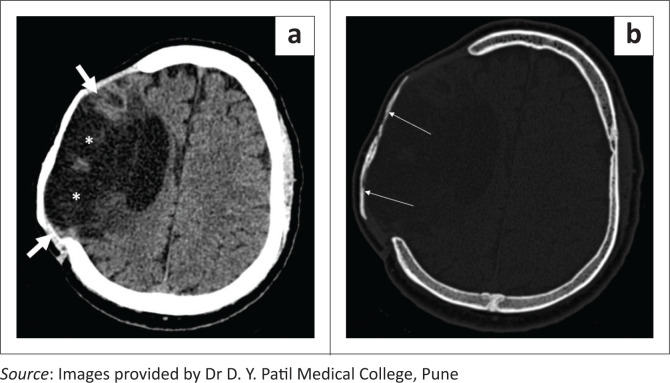
Axial soft tissue (a) and bone window (b) CT brain images in a patient with a right fronto-parietal decompressive craniotomy (a, thick white arrows) demonstrate extracranial herniation of brain tissue (a, asterisks) with thinning and remodeling of the bone flap (b, thin arrows).

**FIGURE 13:**
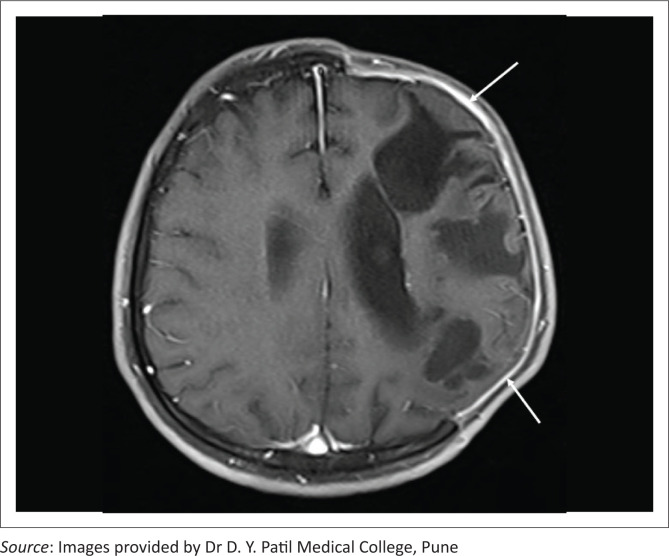
Axial post-contrast T1 weighted MR image in a patient with a left fronto-parietal decompressive craniectomy demonstrates herniation of the brain parenchyma through the craniectomy site along with a thickened enhancing meningogaleal complex (white arrows).

In cases of raised intracranial pressure from any aetiology, such as cerebral oedema or haemorrhage; the oedematous brain parenchyma herniates via the craniectomy defect (Figure 11, Figure 12 and Figure 13). This leads to compression and contusions in the parenchyma at the bony margins of the craniectomy and compression of the superficial cortical veins leading to infarction.^16^

### Tension pneumocephalus

**FIGURE 14:**
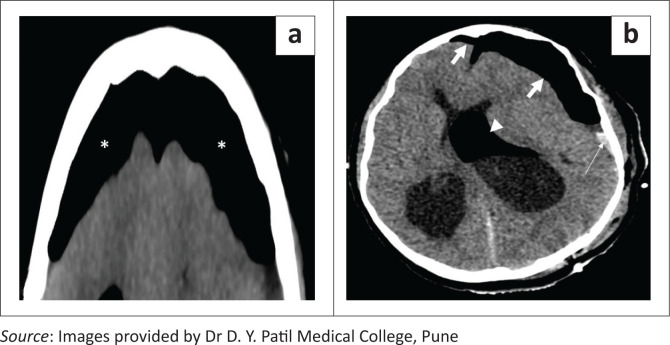
Axial soft tissue window CT image (a) shows tension pneumocephalus in both frontal regions (asterisks) causing mass effect on the underlying brain parenchyma with a heaped-up appearance of the anterior tips of the bilateral frontal lobes (Mount Fuji sign). Axial soft tissue window CT image (b) in a different patient shows asymmetric tension pneumocephalus (thick white arrows) in both frontal regions (left > right), causing mass effect on the underlying brain parenchyma and subfalcine herniation towards right side. There is an associated subdural hemorrhagic collection (thin white arrow) present on the left side and postoperative air in the left lateral ventricle (arrowhead).

Unlike post-operative pneumocephalus, which is an expected finding in the post-operative cranium, tension pneumocephalus is a rare and life-threatening entity. There is build up subdural air that causes compression of the brain parenchyma (Figure 14). It most commonly results from a ball-valve mechanism that causes entry of air into the subdural space.^17^

The diagnosis of tension pneumocephalus is made on CT in the presence of subdural air with the ‘peaking’ sign and the ‘Mount Fuji’ sign (Figure 14a). ‘Peaking’ sign refers to mass effect on the frontal lobes because of the presence of subdural air and the ‘Mount Fuji’ sign refers to separation of the frontal lobes from the falx.^18^

### Subdural and subgaleal hygromas

**FIGURE 15:**
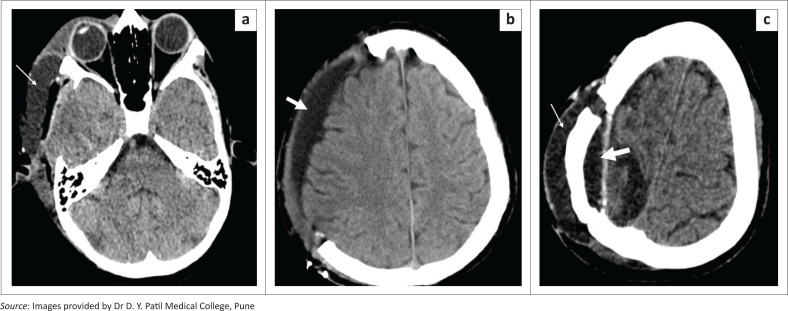
Axial soft tissue window CT image (a) in a patient with a right temporal decompressive craniectomy reveals a subgaleal hypodense fluid collection in the right temporal region (white arrow), suggestive of a subgaleal hygroma. Axial soft tissue window CT image (b) in another patient with a right fronto-parietal decompressive craniectomy demonstrates an extra-axial hypodense fluid collection in the subdural region along the cerebral convexity (white arrow), consistent with subdural hygroma. Axial soft tissue window CT image (c) with a right parietal craniotomy indicating subdural (thick white arrow) and subgaleal (thin white arrow) hygromas.

**FIGURE 16:**
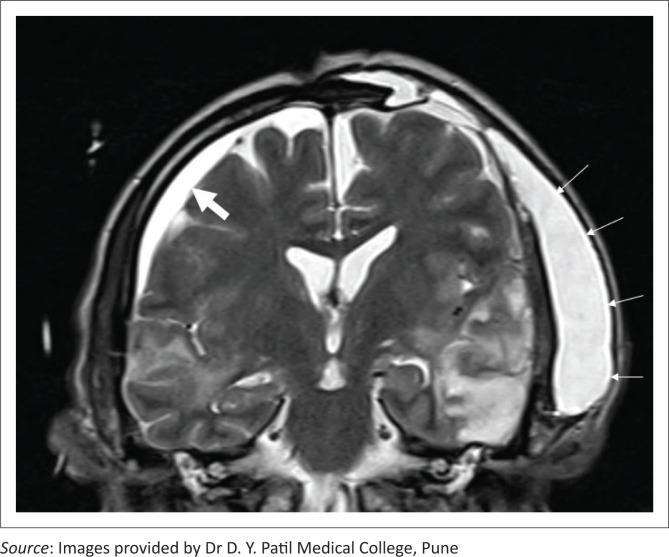
Subgaleal and subdural hygromas. Coronal T2 weighted MRI in a patient with a left parietal craniectomy shows a hyperintense fluid collection in the left subgaleal region, extending beyond the margins of the craniectomy to the left temporal region (thin white arrows). An extra-axial subdural collection is also noted in the right frontoparietal region (thick white arrow).

Subdural and subgaleal hygromas are early complications that usually occur as a result of altered CSF circulation dynamics in the post-operative period leading to CSF accumulation in the subdural or the subgaleal region, most commonly on the side of the surgery.^16^ Rarely, however, they can also be seen on the opposite side or in the interhemispheric region.^19^ The fluid collections accumulate in the first few days following the surgery and resolve spontaneously over weeks or months. They follow CSF attenuation and signal intensity on CT (Figure 15) and MRI (Figure 16) images, respectively and generally do not cause any significant neurological compromise.

### External brain tamponade

**FIGURE 17:**
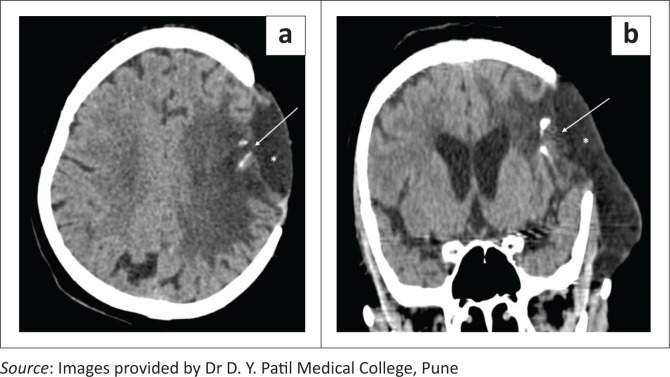
External brain tamponade. Axial (a) and reformatted coronal soft tissue window (b) CT images in a patient with a left fronto-parietal craniotomy demonstrate a large hypodense subgaleal fluid collection causing compression of the underlying left frontoparietal brain parenchyma (long arrows).

External brain tamponade is a rare, life-threatening condition. It is characterised by excessive subgaleal fluid accumulation that exerts pressure on the underlying brain parenchyma. Clinically, the patient presents with neurological deterioration and a tense craniectomy flap. Imaging shows a subgaleal fluid collection producing mass effect on the brain (Figure 17). Midline shift and sub-falcine herniation may be seen in severe cases. Drainage of the subgaleal fluid usually leads to neurological improvement.^20^

### Sinking skin flap syndrome

**FIGURE 18:**
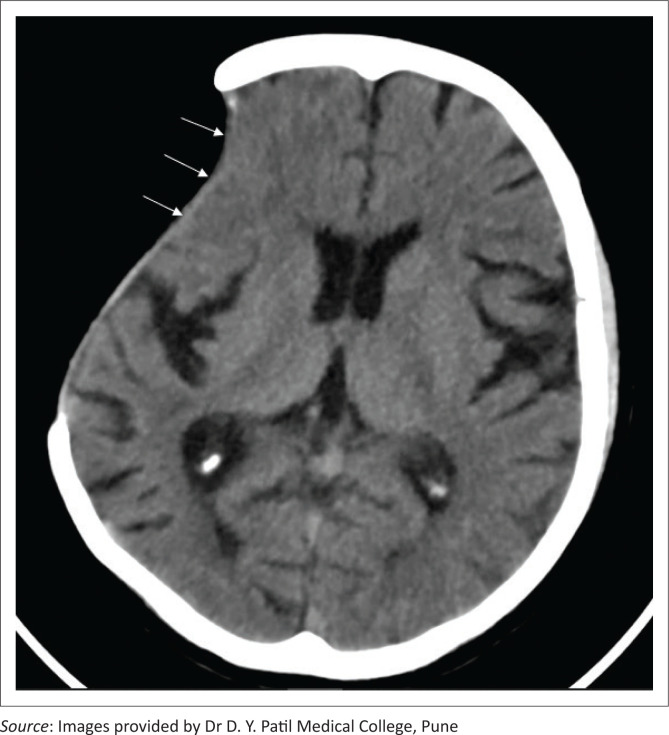
Trephine syndrome. Axial soft tissue window CT brain image reveals a right fronto-parieto-temporal craniectomy with a sunken skin flap (white arrows) and mild deformation of the underlying brain parenchyma without midline shift.

Sinking skin flap syndrome, or Trephine syndrome, is an intermediate to late post-operative complication in patients who undergo craniectomy. There is a sunken appearance of the skin flap and concave appearance of the underlying parenchyma due to chronic exposure of the brain to atmospheric pressure causing deformity of the underlying parenchyma (Figure 18).^21^ Unlike paradoxical herniation, there is no midline shift, subfalcine or uncal herniation. The patients usually present with vague complaints such as headache, dizziness, mood changes and fatiguability.^21^

### Paradoxical herniation

**FIGURE 19:**
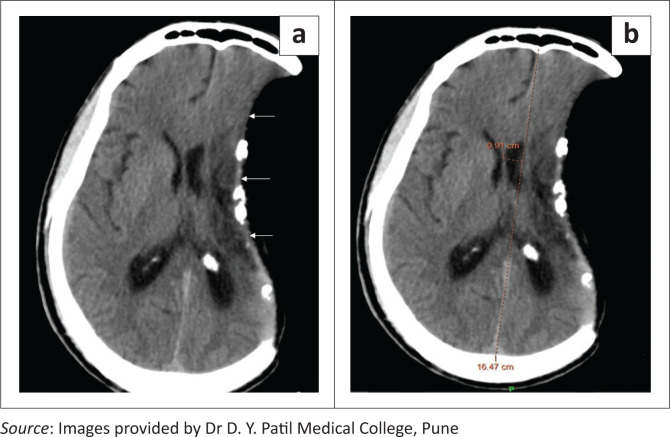
Paradoxical herniation. Axial soft tissue window CT brain images (a, b) reveal a left fronto-temporo-parietal craniectomy with a sunken skin flap (arrows), deformation of the underlying brain parenchyma, midline shift of 9.1 mm (b) and subfalcine herniation to the right side. Associated meningogaleal complex calcifications are also seen.

**FIGURE 20:**
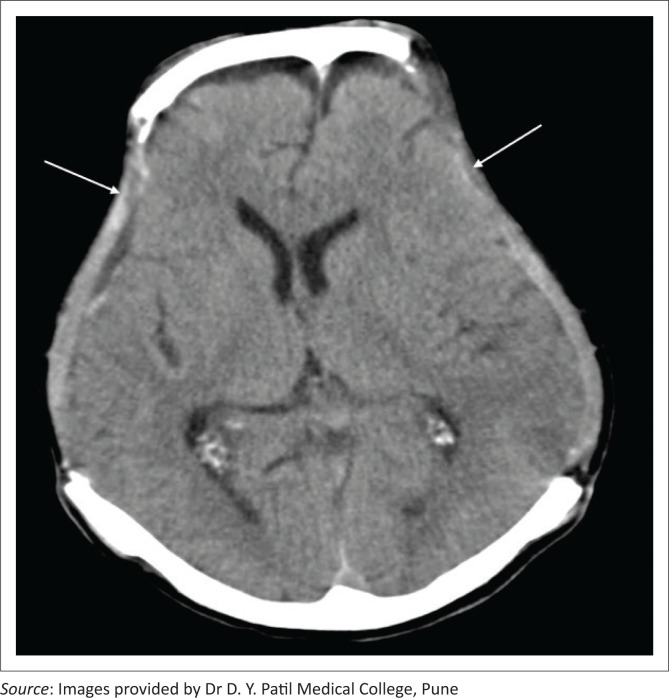
Axial soft tissue window CT image reveals bilateral fronto-parietal craniectomies with sinking of the skin flaps on both sides (left > right) (white arrows) and midline shift towards the right side.

Paradoxical herniation is a rare, late complication in the post-craniectomy patient. It is characterised by a sunken skin flap and contralateral displacement and herniation of brain, with resultant mass effect, midline shift and effacement of CSF spaces (Figure 19 and Figure 20).^20^ The mechanism is usually a decrease in intracranial pressure because of lumbar puncture, ventricular drainage or ventriculoperitoneal shunting, which leads to an imbalance in the intracranial pressure and the atmospheric pressure. It is a potentially life-threatening condition, usually treated by clamping any shunts or drains, putting the patient in the Trendelenberg position, administering fluids and performing an early cranioplasty.^22^

## Conclusion

Imaging plays a crucial role in the assessment of the post-operative cranium, providing valuable information about potential complications and helps in optimising treatment. Radiologists should be familiar with the different early and late complications to provide accurate and timely diagnoses. With proper imaging interpretation, clinicians can make informed decisions regarding patient management, ultimately leading to better post-surgical outcomes and improved patient care.
